# Transatlantic Trade Dispute: Solution for Airbus-Boeing Under Biden?

**DOI:** 10.1007/s10272-021-0947-z

**Published:** 2021-01-26

**Authors:** Stephan Wittig

**Affiliations:** Europa-Kolleg, University of Hamburg, Windmühlenweg 27, 22607 Hamburg, Germany

## Abstract

With the widening of the US measures three weeks before the end of the Trump administration, it falls on Biden to find a solution for the Airbus-Boeing dispute.

Shortly after the US election, the EU announced it would impose countervailing measures on up to $4 billion worth of US aircraft and agricultural products following the World Trade Organization (WTO) arbitration ruling on 13 October 2020 in the Airbus-Boeing dispute against the US. This added another chapter to the 17-year tit-for-tat battle at the WTO. The US had already imposed countermeasures on approximately $7.5 billion of EU exports in late 2019, making this the biggest dispute in the history of the WTO. While hopefully the new US administration signifies a turning point in US-EU trade relations as well as multilateralism and WTO reform, it cannot be expected to turn the clock back four years. Europe, the US and the world have changed tremendously and Trumpism is likely to remain. The chances for a negotiated solution between the US and EU have increased, but it will not be as easy as some in Europe might hope.

## The 1992 US-EU Agreement on Trade in Large Civil Aircraft

To better understand the current situation, it is crucial to understand the origins of this dispute, which dates back to the inception of Airbus in 1970 as a European aviation consortium by France, Germany, Spain and the UK. Government support for aircraft manufacturers first emerged as a contentious issue between Europe and the US in 1988 when Airbus was beginning to eat into Boeing's market with its A320 single-aisle jet – the aircraft type which accounts for almost three-quarters of all planes sold. Subsequent proceedings at the WTO's predecessor General Agreement on Tariffs and Trade (GATT) resulted in a bilateral agreement in 1992 between the US and the EU[1] on Trade in Large Civil Aircraft (TLCA), which regulated the permitted levels of support for the American and European producers of wide-body civil aircraft (Wittig, 2010).

1 European Communities (EC) at the time.

**Stephan Wittig**, University of Hamburg, Germany.

Addressing European measures, direct government support was fixed to a maximum of 33% of total development costs, and loans had to be provided with an interest rate covering at least the government's loan costs. Production subsidies were prohibited. Addressing US support, indirect state aid was limited to a maximum of 3% of the commercial aircraft industry's annual turnover, or to a maximum of 4% of each company's turnover in civil aviation. A precise definition of indirect aid, however, was never agreed upon.

In the autumn of 2004, trade representatives from the EU and the US engaged in negotiations in an attempt to modify the agreement, but those negotiations failed (Carbaugh and Olienyk, 2007). Thereupon, the US unilaterally withdrew from the TLCA initiating this 17-year saga by filing a suit at the WTO. This occurred at the same time that Airbus launched the A380 and A350 projects and Boeing was about to lose its leading position in the market for airplanes with more than 100 seats. In 2003, Airbus had delivered more aircraft than Boeing for the first time (Figure 1).

The fierce battle for this market is not surprising, as global airline traffic was forecasted to more than double over a 20-year period, leading to a demand for up to 25,000 commercial aircraft representing a market value of around $2 trillion at the time (Boeing, 2004).[2] After the US realised that its expectations of stabilising market shares with the TLCA had been off base, its withdrawal from the agreement in 2004 came as no surprise.

## The Airbus-Boeing disputes at the WTO

The tit-for-tat began with the US requesting WTO proceedings against the EU on 6 October 2004 (DS 316). On the same day, the EU followed suit and brought a claim against the US (DS 317, later DS 353).

In its filing, the EU claimed that Boeing had received over $19.1 billion in illegal subsidies from state, local and federal sources. The US in particular contested the so-called launch aid of approximately $15 billion granted to Airbus by its consortium states. Launch aid[3] consists of repay-

2 The market potential 2004-2023 was estimated by Boeing to be 25,000 new commercial airplanes worth around $2.0 trillion (in 2003 US dollars).

3 Launch aid was the term used by the US, the term member state financing was used in Europe. In this paper, only the term launch aid will be used.

Figure 1
**Total aircraft deliveries by manufacturer (1974-2020)**

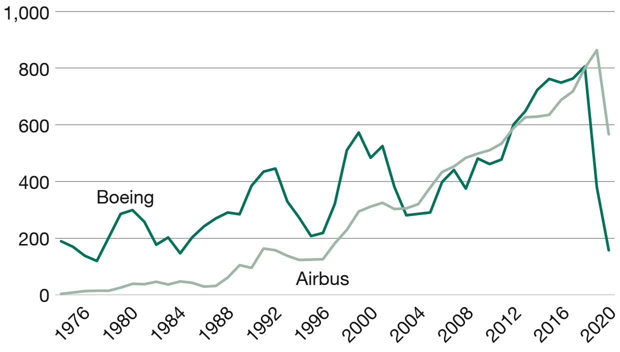

Note: The sharp drop in Boeing deliveries in 2019 is due to the grounding orders of Boeing's 737 Max. In March 2019, airworthiness certificates for the 737 Max had been withdrawn around the globe following two crashes. The 737 accounted for 72% of airplanes delivered by Boeing in 2018.

Sources: Airbus Summary Results, 1989-2018; Airbus annual reports; Boeing Orders and Delievery Database.

able, low-interest rate loans, which were structured according to the provisions of the bilateral TLCA of 1992. However, the TLCA was not applicable anymore due to the US' withdrawal.[4] Therefore, both parties claimed violations of the WTO Agreement on Subsidies and Countervailing Measures (SCM) and of the GATT 1994.

4 See, e.g. the WTO AB report of 18 May 2011 for 'European Communities – Measures Affecting Trade in Large Civil Aircraft', WT/DS316/AB/R, p. 617.

Both disputes were heard in parallel by different WTO Panels (Figure 2). As these became the two largest disputes in WTO-history, the regular timelines for panel and Appellate Body (AB) reports could not be met. The WTO issued the panel report in the Airbus case five years later, in June 2010, and in the Boeing case in March 2011. The decisions on the respective appeals were also nine to ten months apart: The AB report in the Airbus dispute was issued in May 2011, the Boeing report in March 2012.

## WTO findings in the Airbus dispute

WTO Panels engage in fact finding and review the factual as well legal aspects of the case, whereas the WTO AB only reviews issues of law and legal interpretations developed by the panel. The AB in the Airbus dispute (DS 316) reversed some of the key findings of the panel, notably that launch aid would constitute a prohibited export subsidy – the AB decided that launch aid falls into the category of actionable subsidies.[5] This is rather central because export subsidies are per se prohibited and have to be re-

5 WT/DS316/AB/R, p. 609.

Figure 2
**Timeline of the parallel WTO proceedings**

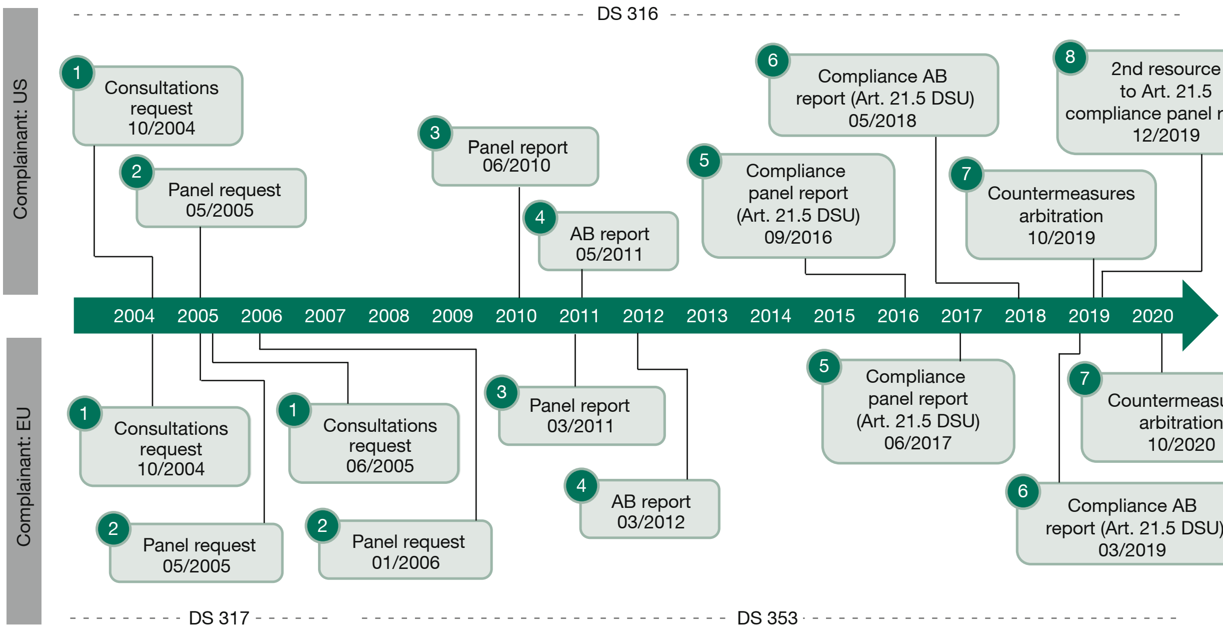

Source: Author's own illustration; data based on WTO Dispute Settlement portal.

Figure 3
**Overview on the WTO Agreement on Subsidies and Countervailing Measures**

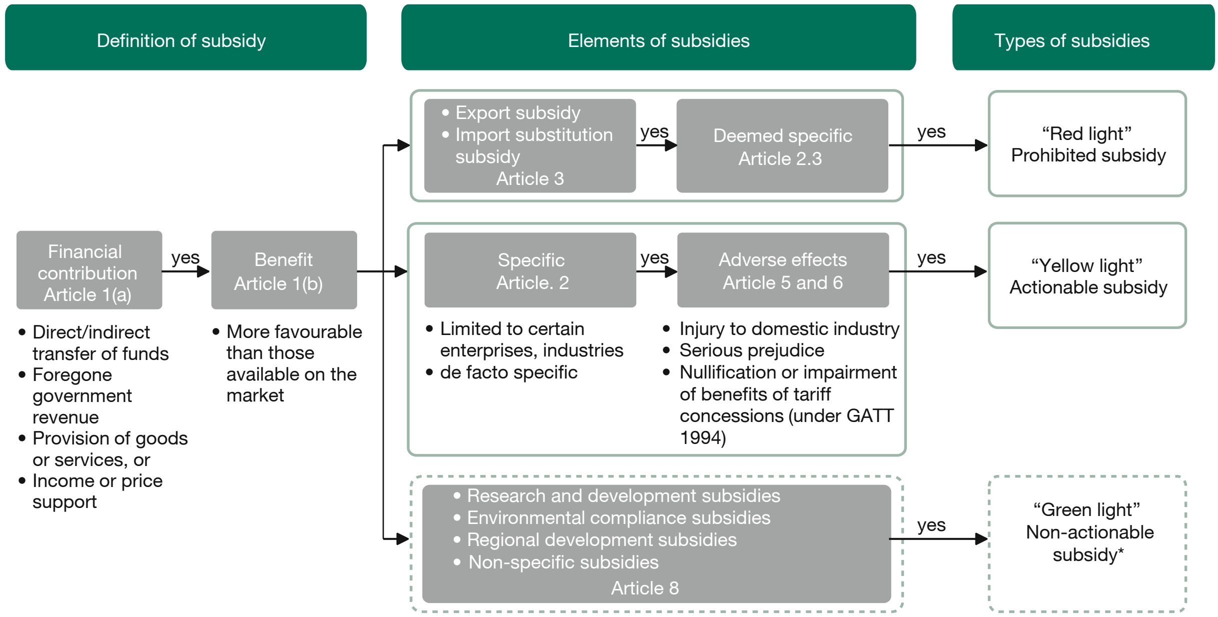

Note: *The provision on non-actionable subsidies expired in 2000.

Source: WTO SCM Agreement.

moved, whereas specific subsidies are only "actionable" with much softer remedies.

In the WTO SCM Agreement, there are three categories of subsidies: prohibited, actionable and non-actionable subsidies.[6] As shown in Figure 3, in general, a financial contribution has to be proven as well as a benefit for the subsidy recipient, i.e. more favourable conditions than available on the market. For actionable subsidies, it has to be proven that they are (a) specific (e.g. enterprise or industry-specific, or de facto specific), and (b) cause adverse effects (e.g. an injury to a domestic industry or serious prejudice to a WTO member state).

There were five areas that were mainly contested by the US. Subsidies allegedly granted by the EU and certain member states: launch aid contracts, loans by the European Investment Bank, infrastructure-related measures, corporate restructuring measures (debt forgiveness, equity infusions and grants), and research and development funding (see Table 1).

The AB reversed the panel's finding that the A380 launch aid qualified as prohibited export subsidies.[7] However, the AB upheld the finding of the panel that each instance of launch aid is a specific subsidy. Also, almost all infrastructure measures as well as German and French restructuring measures were found to be specific subsidies. The contested research grants were found to be specific subsidies, but not found to cause adverse effects or to constitute serious prejudice to the US.

The AB upheld the panel's finding, however, that serious prejudice to the US' interests[8] was caused by specific subsidies such as the launch aid measures, certain infrastructure measures and equity infusions (Wittig, 2012; Kinestra, 2012).[9] The remedy for such actionable subsidies is that the member state "shall take appropriate steps to remove the adverse effects or shall withdraw the subsidy"[10] – which the AB requested from the EU.[11]

It is important to note that neither the initial panel nor the AB quantified the specific subsidies or the adverse ef-

6 Non-actionable subsidies expired in 2000 and have not been renewed since.

7 WT/DS316/AB/R, p. 479, 609-610, para. 1414 (j).
8 Within the meaning of Article 5(c) SCM.
9 WT/DS316/AB/R, p. 594, 605.
10 Article 7.8 SCM.
11 WT/DS316/AB/R, para. 1416.

Table 1
**WTO Panel and Appellate Body (AB) findings in the Airbus case**

| Subsidy | Description | Panel / AB decision |
|---|---|---|
| Launch aid (LA) / Member state financing | • France, Germany, Spain and the UK granted loans for the development and launch of new aircraft models in accordance with the TLCA<br>• In alignment with the bilateral TLCA of 1992, government support was fixed to a maximum of 33% of the total development costs, and the loans had to be provided with an interest rate at least covering the loan costs of the government, and for a maximum of 17 years | • The AB reversed the panel's finding that A380 LA measures qualified as prohibited export subsidies<br>• However, the AB upheld the panel's finding that each instance of LA is a specific subsidy<br>• The AB upheld, although narrower in scope, that LA caused serious prejudice |
| European Investment Bank (EIB) loans | • Through partial funding of investment projects (up to 50% of the purchase price), EIB funded the renewal of passenger aircraft fleets, which was usually accompanied by capacity expansions<br>• In 2000-06, some 61% of EIB aircraft acquisitions financed were Airbus planes and the remainder from other manufacturers (24% Boeing) | • The panel found that none of these subsidies were specific under Article 2 SCM |
| Infrastructure grants | • The provision of certain infrastructures, e.g. the Mühlenberger Loch industrial site in Hamburg, the extension of the airport runaway in Bremen and the Aéroconstellation industrial site in Toulouse<br>• The disbursement of certain infrastructure-related grants by various regional authorities in the EU | • The AB reduced the number of infrastructure measures constituting specific subsidies, but e.g. the Mühlenberger Loch industrial site in Hamburg was still considered a specific subsidy causing serious prejudice |
| Corporate restructuring measures | • In the 1990s, the German and French government supported the restructuring of the respective national consortium companies, such as Deutsche Airbus, MBB, Aérospatiale, Dassault Aviation etc., e.g. in 1989 with a KfW capital contribution into Deutsche Airbus and its 1992 transfer of shares | • The AB reduced the number of corporate restructuring measures constituting specific subsidies: e.g. the French government's transfer of its 45.76% stake in Dassault Aviation to Aérospatiale is no longer considered a specific subsidy |
| Research funding | • The EU pursues framework programmes for research and technology development which serve mostly to subsidise technologies at the pre-competitive stage<br>• Also Airbus Consortium States provided research funding, e.g. through the German aviation research programme (LuFo) | • The framework programmes and member states' grants were found to be specific subsidies, but not to cause adverse effects or to constitute serious prejudice |

Source: Author's own illustration; data based on WTO Dispute Settlement portal.

fects for the US.[12] Overall, it can be summarised that the panel report already held several positive findings for the EU, but the AB report improved its position significantly (Chianale, 2013, 328).

## WTO findings in the Boeing dispute

The twin case against the US ran in parallel to the case against the EU. The decisions in the US dispute were lagging 9 to 12 months behind the EU case (see Figure 2) because the EU had requested a new proceeding in June 2005 (DS 353) with a broader scope than the original case (DS 317).

The panel report found that Boeing[13] had received prohibited and actionable subsidies from various sources:

1. the US Government through prohibited "Foreign Sales Corporation" (FSC) export subsidies,
2. NASA and the US Department of Defence through research and development programmes and general support,
3. the States of Washington, Kansas and Illinois through tax breaks and other programmes.[14]

Although the WTO Panel did not uphold all claims by the EU, the panel estimated the total amount of specific subsidies received by Boeing between 1989 and 2006 to have been at least $5.3 billion (see Table 2).

The panel found that the FSC-related subsidies provided to Boeing constituted prohibited export subsidies.[15] The finding was not appealed by the US, as it had already lost previous disputes on FSC.

Similar to the panel, the AB found that the measures by NASA and the Defence Department had enabled Boeing

12 For example, the AB did not quantify the benefit from the launch aid granted to Airbus, see WT/DS316/AB/R, p. 608; and Wittig (2012, 25).
13 In this paper, "Boeing" stands for The Boeing Company and the McDonnell Douglas Corporation prior to its merger with Boeing – as applied in the WTO Panel Report.
14 WTO Panel Report: United States – Measures Affecting Trade in Large Civil Aircraft (DS 353), WT/DS353/R, p. 584.
15 Pursuant to Article 3.1(a) SCM. See WTO dispute DS108, US – Tax Treatment for 'Foreign Sales Corporations' and following Article 21.5 DSU proceedings.

Table 2
**US subsidies found by the WTO Panel (DS 353)**

| Government or granting authority | Measures found to be specific subsidies by the WTO Panel | Subsidy amount |
|---|---|---|
| US Government | • Tax exemptions and tax exclusions provided under Foreign Sales Corporation legislation and Extraterritorial Income Exclusion Act, including the transition and grandfather provisions | $2.1 billion |
| NASA | • Payments made to Boeing pursuant to procurement contracts entered into under eight aeronautics research and development programmes<br>• Access to government facilities, equipment and employees provided to Boeing pursuant to procurement contracts and Space Act Agreements | $2.6 billion |
| US Department of Defence | • Payments made pursuant to assistance instruments entered into under 23 Research, Development, Test and Evaluation (RDT&E) programmes<br>• Access to government facilities provided to Boeing pursuant to assistance instruments entered into under the RDT&E programmes | Not quantified by WTO (*est. $0.3-$2.4 billion*) |
| State of Kansas (and municipalities therein) | • Property and sales tax abatements provided to Boeing pursuant to Industrial Revenue Bonds issued by the State of Kansas and municipalities therein | $0.48 billion |
| State of Illinois (and municipalities therein) | • Reimbursement of a portion of Boeing's relocation expenses<br>• 15-year tax credits and abatement of property taxes<br>• Payment to retire the lease of the previous tenant of Boeing's new headquarters building | $0.01 billion |
| State of Washington (and municipalities therein) | • Business and Occupation (B&O) tax reduction provided for in Washington House Bill 2294 (HB 2294), as well as City of Everett B&O tax reduction<br>• Tax credits for preproduction development, software, hardware and property taxes (HB 2294)<br>• Workforce development programme and employment resource centre | $0.08 billion (*future benefits not included*) |
| | Total of at least $5.3 billion | |

Source: WTO Panel Report DS 353, WT/DS353/R, p. 584.

to launch its technologically advanced 787 in 2004,[16] and agreed with the panel's statement that the

> ability to define and manage the complex interaction of design processes, organization and tools so as to enable the robust development and manufacturing of an aircraft at minimum time and cost [...] is a challenge that Boeing can meet thanks in large part to NASA and USDOD funding.[17]

Furthermore, the AB found that these measures caused serious prejudice – in particular, a significant loss in sales for Airbus in several markets,[18] a threat of displacement and impedance,[19] and significant price suppression with respect to the 200-300 seat LCA market.[20]

The tax benefits provided to Boeing were found to cause serious prejudice, albeit limited by the AB to significant lost sales in the 100-200 seat LCA market.[21] With respect to the tax benefits, it is important to note that only benefits up until 2006 were included in the panel's estimate of around $500 million. The EU estimated the value of the Washington State subsidies alone to be at least $3.56 billion from 2004 to 2024.[22]

In terms of remedies for the FSC subsidies, which were prohibited, the panel refrained from making any new recommendations under Article 4.7 SCM. The recommendation to withdraw the prohibited measures without delay remained operative from prior FSC-cases, which the AB upheld.[23] The AB also upheld the panel's recommendation pursuant to Article 7.8 SCM that the US should take appropriate steps to remove the adverse effects of the other actionable subsidies found or to withdraw the subsidies.[24]

With a closer look at those twin disputes, it becomes apparent that the AB went further in the Boeing dispute in its findings than the panel. In contrast, the AB reversed substantial findings of the panel in the Airbus dispute (Kaienburg, 2014, 144).

16 WT/DS353/AB/R, para. 1350(d)(i)(A)(1).
17 WT/DS353/AB/R, para. 1350(d)(i)(A)(2).
18 Namely, Australia, Iceland, Kenya and Ethiopia. See WT/DS353/AB/R, para. 1350(d)(i)(A)(4).
19 In this instance in Australia, see WT/DS353/AB/R, para. 1350(d)(i)(A)(5).
20 WT/DS353/AB/R, para. 1350(d)(i)(A)(6).
21 WT/DS353/AB/R, para. 1349.
22 WT/DS353/R, p. 199.
23 WT/DS353/AB/R, para. 1352, and footnote 2716. US – FSC under Article 4.7 of the SCM Agreement continues to be 'operative', referring to the US – FSC (Article 21.5 – EC II) panel report, para. 8.2; and to AB Report, US – FSC (Article 21.5 – EC II).
24 WT/DS353/AB/R, para. 1352.

## Enforcement at the WTO

Some authors have argued that the WTO was not the right forum to address this transatlantic trade dispute in the first place (e.g. Kaienburg, 2014, 145). But the WTO actually managed handling two parallel cases of this magnitude fairly well. However, a systemic weakness also became apparent in these disputes – the panel/AB "recommends that the [respondent] takes appropriate steps to remove the adverse effects found to have been caused by its use of subsidies, or to withdraw those subsidies".[25]

One of the major drawbacks of the WTO dispute settlement system – at least compared to national legal systems – constitutes the fact that sanctions, if any, are never retroactive. Only if a challenged action remains in place after an adverse panel ruling, the WTO Dispute Settlement Body (DSB) may allow the aggrieved party to withdraw concessions.[26] According to Article 22.4 DSU, the suspension of concessions shall be substantially equivalent to the ongoing harm suffered from the violation (Wolfrum et al., 2006; Pauwelyn, 2010). Furthermore, Article 20 SCM implicitly states that the WTO system does not allow for retroactive or even punitive damage compensation in subsidies cases.[27]

As these proactive countervailing measures may only be imposed after a "reasonable period of time", which is usually up to 15 months pursuant to Article 21.3 DSU, the defending parties have a strong incentive to delay panel proceedings.[28] The violation and the associated benefits can basically remain in place unhampered for the duration of the proceedings. Evidence of this – besides the length of the disputes of one to four years – is the relatively high appeal rate of 66% (WTO, 2020, 195). In addition to political economy reasons to fight hard for the challenged measures, it allows for the further delay of the implementation of remedies such as countervailing duties.

## Eight years of compliance battle at the WTO

The same thing happened in the Airbus-Boeing saga, where the fierce legal battle continued for another eight years (see Figure 2). In the Airbus case, the US requested consultations regarding compliance pursuant to Article 21.5 DSU – just days after the EU had notified the DSB that it had taken appropriate steps to bring its measures into conformity with its WTO obligations, and to comply with the AB's recommendations.[29] The EU raised serious systemic concerns that – despite its compliance report – the US had already made a request for the authorisation of countermeasures,[30] along with its request for compliance consultations.

In April 2012, an Art 21.5 DSU compliance panel was established in the Airbus case. Given the complexity of the dispute, the panel report was postponed year by year until it was circulated in September 2016. The panel concluded that the US failed to demonstrate that the A380 and A350 launch aid constituted prohibited export subsidies, but found that the EU had failed to implement recommendations of the DSB. In turn, the EU and US appealed the compliance panel report.

In its May 2018 decision, the AB found that the subsidies in the single-aisle market had expired before 1 December 2011 – the deadline for the EU to comply with the recommendations of the original dispute.[31] However, the AB upheld that subsidies existed in the post-implementation period with respect to the twin-aisle market, i.e. finding that the EU did not comply with the ruling in that market segment. Subsequently, the US requested that the arbitrator resume its work to authorise countermeasures.[32] In turn, the EU requested the establishment of a second compliance panel, which issued its report in December 2019.[33] The EU appealed due to similar findings; the AB panel is currently pending.

The Boeing dispute saw a similar battle over compliance with the initial ruling. The EU requested compliance consultations and the matter was referred to the original panel in October 2012.[34] The compliance report of 2017 was subsequently appealed by both sides until the final AB compliance report was released in March 2019. The AB found, inter alia, that the US had not withdrawn FSC/ETI subsidies for Boeing in the post-implementation period.[35] Furthermore, the AB agreed that Boeing was able to use the benefits of the Washington State business and

25 For example, the United States in DS 353. See WT/DS353/AB/R, para. 1352.
26 The defendant WTO member has a 'reasonable period of time' – usually up to 15 months – to bring its policies into conformity with its WTO obligations after it has been found to violate them, see Article 21.3 (c) DSU.
27 Although it is discussed in the literature whether in the case Australia – Automotive Leather, DS 126, some sort of retroactivity has been introduced. See, e.g. Matsushita et al. (2006, 185-187) or Wolfrum et al. (2009).
28 The WTO AB seems to enjoy an extensive discretion on this. See Matsushita et al. (2006); for a discussion of "reasonable period of time", see also Davey (2005).
29 See WTO summary of the dispute (DS 316).
30 Namely the suspension of concessions pursuant to Article 22.2 DSU.
31 WT/DS316/AB/RW, p. 267.
32 The initial request for the authorisation under Article 22 DSU was made in December 2011, but due to the Article 21.5 DSU compliance panel, the EU and US requested in January 2012 to suspend the Article 22 DSU arbitration proceeding until either party requests their resumption. See WTO summary of the dispute (DS 316).
33 See WT/DS316/ARB, p. 15, and WT/DS316/RW2.
34 See WTO summary of the dispute (DS 353).
35 To the extent that Boeing remained entitled to FSC/ETI tax concessions. See WT/DS353/AB/RW, p. 176.

occupation (B&O) tax rate reductions to lower prices in particularly price-sensitive sales campaigns in the single-aisle LCA market,[36] causing significant lost sales, and a threat of impedance.[37]

In May 2020, the US informed the DSB of its compliance and that the State of Washington had enacted legislation in March 2020 to remove the preferential B&O tax rate for aerospace manufacturing, retailing and wholesaling. The EU objected and requested the DSB arbitrators in June 2020 to authorise countermeasures.[38]

## Largest WTO awards in history

With a combined annual value of around $11.5 billion, the awards in this transatlantic trade war are the biggest in WTO history (see Figure 4). On 2 October 2019, the US received the right to impose countermeasures on up to $7.49 billion annually worth of European exports.[39] Similarly, the WTO Arbitrators gave the EU permission for countermeasures on US exports of up to $3.99 billion annually on 13 October 2020.[40]

Figure 4
**Highest WTO arbitration awards**

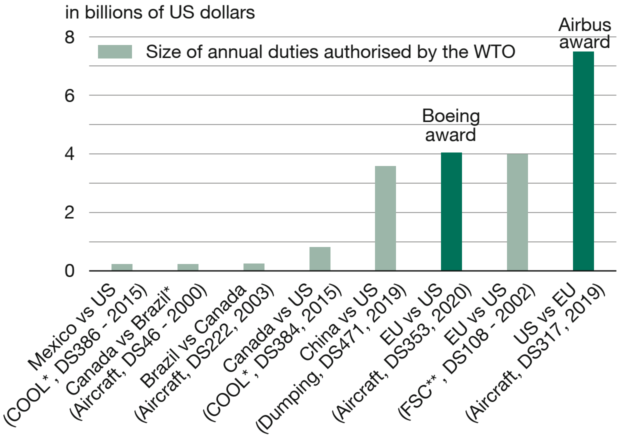

Note: *Certain Country of Origin Labelling Requirements; **Foreign Sales Corporation.

Source: WTO documents WT/DS386/ARB, p. 80; WT/DS46/ARB, p. 27; WT/DS222/ARB, p. 33; WT/DS384/ARB, p. 80; WT/DS471/ARB, p. 67; WT/DS108/ARB, p. 33; WT/DS353/ARB, p. 121; WT/DS316/ARB, p. 156.

Shortly after the WTO decision, the US imposed countermeasures on $7.5 billion of imports from the EU, instituting a 10% tariff on large civil aircraft and a 25% tariff on certain other products, specifically targeting the Airbus consortium states France, Germany, Spain and the UK.[41] This was done on product groups with a high pain point, e.g. French wine and cheese, Scotch whiskey, Spanish olives, German wine and industrial products. The duties on aircrafts were increased from 10% to 15% in March 2020.

Due to the COVID-19 pandemic, the award for the EU had been delayed by several months, weakening the EU's negotiation position as the US measures were already in place. In November 2020, the EU announced it would mirror the US duties and impose a 15% tariff on large civil aircraft, and 25% duties on politically sensitive industries for Trump and his Republican allies in Congress, including US agricultural products, such as ketchup, rum, vodka, nuts, but also tractors, coal and video games (Stearns, 2020).

## First steps towards settlement

Throughout the 17-year battle, there had been several attempts made by the EU to find a negotiated solution. For instance, in July 2019 – before the WTO awards were released – the EU submitted a proposal for a new bilateral regime on limiting aircraft subsidies, including a mechanism for transatlantic monitoring and dispute settlement, as well as a proposal on how to better address aviation subsidies in the WTO framework (BDI, 2020). The US never reacted to the proposal, but publicly demanded the repayment of subsidies by Airbus, which the EU refused with reference to WTO law – there is no retroactivity in WTO law.

Of course, the Airbus-Boeing dispute must be examined within the broader context of the transatlantic trading relationship as well, which significantly deteriorated under Trump. For instance, Trump imposed aluminium and steel tariffs due to 'national security' under Section 232 in March 2018.[42] At the beginning, the US exempted partners such as Canada, Mexico and the EU, but this exemption was not extended. Instead of turning the EU and Canada into allies in his trade fight against China,

36 The AB also found that the panel "was not required to establish that the per aircraft amount of the subsidies available for these sales campaigns exceeds the differentials in the net prices of Airbus' and Boeing's competing aircraft" (WT/DS353/AB/RW, 179).
37 In relation to five particularly price-sensitive campaigns in the single-aisle market.
38 See WTO dispute summary (DS 353).
39 The Arbitrators determined the monetary values for impedance and lost sales during the 25-month reference period of December 2011-2013 (which was the same reference period used in the compliance proceedings). See WT/DS353/ARB, p. 121.
40 WT/DS316/ARB, p. 156.
41 USTR Notice of Determination and Action Pursuant to Section 301: Enforcement of U.S. WTO Rights in Large Civil Aircraft Dispute, US Federal Register, Vol. 84, No. 196, notice 54245, 9 October 2019.
42 The US introduced a 25% tariff on steel imports, and a 10% tariff on aluminum in March 2018. See the Executive Order of 8 March 2018, Proclamation 9704 and 9705, US Federal Register, Vol. 83, No. 51, 15 March 2018.

Trump alienated them. The EU and Canada together export seven times as much to the US as China, so the tariffs have a much larger impact on them.[43] In turn, the EU started WTO proceedings against the US,[44] and imposed safeguard measures in alignment with WTO law to offset the negative effects of US tariffs.[45] Among other measures, President Trump also threatened to impose tariffs on cars, which EU Commission President Juncker prevented during a visit to the White House in July 2018 (European Commission, 2018b).

With respect to the Airbus-Boeing dispute, Trump threatened to “strike back even harder”, potentially raising tariffs to the allowed maximum of 100%, if the EU were to impose tariffs on US products – that was in October 2020 after the WTO released its award of $4 billion in favour of the EU (Bashuk and Horobin, 2020). That might have been one of the reasons why the EU waited with its decision to actually impose the WTO-permitted countervailing duties until the outcome of the US election was relatively certain – namely on 9 November.

Only after the mirroring of US tariffs by the EU did the US start to engage in somewhat serious negotiations to resolve the Airbus-Boeing dispute. US Trade Representative Robert Lighthizer, EU Trade Commissioner Valdis Dombrovskis and French and German officials were in regular contact in November and December 2020. Dombrovskis even publicly announced that reaching an agreement would still be possible before Trump left office (Stearns and Edwards, 2020). Reasons for the US’ change of attitude – besides the EU’s duties – might have been a personal interest in solving the biggest trade dispute in WTO history before the end of the term, but also Boeing’s current business situation.

Even before the pandemic and its effects on air travel hit, Boeing experienced manufacturing issues and a massive drop in revenues due to the grounding of the 737 Max in March 2019. Plane deliveries fell by 81% from 806 in 2018 to 157 in 2020 (see Figure 1). Also, Boeing experienced massive order cancellations in 2020 with net orders down by 1,026 planes, whereas Airbus proved to be more resilient during the crisis and increased its net orders by 268 (Hemmerdinger, 2021). In addition, Boeing took another financial hit on 7 January 2021 with a $2.5 billion-settlement with the US Department of Justice regarding criminal charges that followed the two deadly crashes of the 737 Max. However, this amount was even considered “low” by financial analysts and US Senator Richard Blumenthal called the deal struck during the waning days of the Trump administration a “disgrace” for letting Boeing off the hook too easily (Johnson and Levin, 2021).

The Airbus-Boeing negotiations with the Trump administration came to an end when the US unilaterally extended its tariffs to other product groups on 30 December 2020. The goods affected include aircraft manufacturing parts, e.g. Airbus wings and components, but also certain wines and spirits from France and Germany. Before that, Airbus was still able to deliver planes to its US customers without having to pay the 15% tariff because Airbus produces planes in a plant in Alabama. Boeing does not assemble aircraft in Europe and therefore has no option to avoid the tariffs, which customers such as Ryanair have said they would be unwilling to pay.

## Trade priorities of the Biden administration

With the widening of the US measures three weeks before the end of the Trump administration, it falls on Biden to find a solution for the Airbus-Boeing dispute. The tariffs on exports worth $11.5 billion distort transatlantic trade and hurt consumers as well manufacturers on both sides of the Atlantic. Previous aircraft WTO cases, such as the Canada-Brazil disputes over subsidies for their respective aircraft manufacturers Bombardier and Embraer, should show both sides that a solution needs to be found politically and not in court. The US and the EU have both been found to be violating WTO rules. It is time to end the 17-year trade war, which has taken up substantial resources on both sides.

But the Biden administration will have to deal with a wide range of trade topics. Trump alienated a lot of former US allies with his ‘America First’ approach. Many are looking forward to the US reassuming its leadership role and returning to a more rules-based multilateralist approach. But it will not just be turning back the clock four years – Europe, the US and the world have changed tremendously, and Trumpism is likely to remain. Some of Trump’s rhetoric resonated and public opinion has shifted towards more economic nationalism. Therefore, it is expected that Biden will only partly deviate from the protectionism of the Trump era. There is, for example, a strong lobby for the aluminium and steel tariffs by unions and parts of the Democratic Party. A compromise might be a reduction in tariffs for allies, or to move away from the highly disputed grounds for these tariffs, which were imposed due to ‘national security’ concerns under section 232.

43 In 2017, the EU exported $12.7 billion to the US, Canada $7.7 billion, compared to China with $2.9 billion (Bown, 2018; Long, 2018).

44 The EU requested consultations at the WTO in June 2018, the panel was composed in January 2019. See WTO dispute DS 548, United States – Certain Measures on Steel and Aluminium Products.

45 The EU imposed safeguard duties on approximately €2.4 billion of US exports, strategically targeting ‘iconic’ products such as jeans, Bourbon whisky and motorbikes (see European Commission, 2018).

Similar to the Obama administration, which had to deal with the aftermath of the financial crisis, Biden will have to focus on pressing domestic issues at the beginning of his term, such as fighting the coronavirus pandemic and its consequences and trying to heal the deep divisions within the country. It took Obama two years to address trade issues. Biden, however, is determined to strengthen democratic alliances and to repair relationships with global trading allies. The US has lost significant political capital abroad, e.g. with its withdrawal from TPP and the Paris climate agreement and by blocking the nomination of new WTO AB members.

The AB has not been able to pursue any work since the expiration of the terms of two of the last three remaining judges in December 2020. WTO AB reform was already a contentious issue under the Obama administration, and it remains unclear whether this will be resolved quickly. A first step towards regaining trust would be to find a consensus for a new WTO Director General – a position that has been vacant since last summer due in part to a US blockade. For President Biden, it will be important to regain trust with close allies such as the EU and Japan, and to find a common approach to pressing trade issues, e.g. digital taxes, WTO reform and how to address China and its state-owned enterprises, forced technology transfer and market distortions. Progress is also vital within the Trilateral Initiative of the US, EU and Japan regarding industrial subsidisation.

It will take time for Biden's USTR nominee Katherine Tai to be approved and for her team to be fully operational. Optimists believe the Airbus-Boeing dispute could be settled within the first six to nine months of the new administration – despite the rather substantial gap between the WTO awards of $7.5 and $4 billion respectively. Though it has to be noted that an EU-compliance appeal is still pending and that the last A380s will be delivered this year, which is why it has been argued that the US award should be lowered by at least $2 billion. The EU suggests a temporary suspension of the tariffs during the negotiation period.

The major question will be whether both sides will aim for a bilateral agreement or a broader scope. In an ideal world, aircraft subsidies would be agreed upon with all major players in the field, i.e. Brazil, Canada, China and Russia, to name the most important. For instance, the old plurilateral GATT and later WTO Agreement on Trade in Civil Aircraft could provide a framework that the parties could fill with an agreement on subsidies in the aviation sector. But such a plurilateral solution to level the playing field is ambitious and would certainly take longer than a bilateral approach. The two parties must first come to an agreement on a way out of this transatlantic aircraft battle. Despite its much longer life cycles and high entry barriers, the aviation world has also changed in the past 17 years – a big leap forward will be needed for the next generation of aircrafts and engines, and addressing Comac and China's massive subsidies should be a major concern. The aerospace industries on both sides of the Atlantic are struggling; the COVID-19 pandemic could thereby create a positive momentum for a negotiated solution and a revival of the transatlantic alliance. But despite all optimism and an anticipated change in tone, Europe should not expect the Biden administration to be less tough on the substantive matters of the dispute.
